# The Hyaluronan/CD44 Axis: A Double-Edged Sword in Cancer

**DOI:** 10.3390/ijms242115812

**Published:** 2023-10-31

**Authors:** Nicola Cirillo

**Affiliations:** Faculty of Medicine, Dentistry and Health Sciences, The University of Melbourne, 720 Swanston Street, Carlton, VIC 3053, Australia; nicola.cirillo@unimelb.edu.au

**Keywords:** hyaluronic acid, CD44, cancer stem cells, chemoresistance, carcinogenesis

## Abstract

Hyaluronic acid (HA) receptor CD44 is widely used for identifying cancer stem cells and its activation promotes stemness. Recent evidence shows that overexpression of CD44 is associated with poor prognosis in most human cancers and mediates therapy resistance. For these reasons, in recent years, CD44 has become a treatment target in precision oncology, often via HA-conjugated antineoplastic drugs. Importantly, HA molecules of different sizes have a dual effect and, therefore, may enhance or attenuate the CD44-mediated signaling pathways, as they compete with endogenous HA for binding to the receptors. The magnitude of these effects could be crucial for cancer progression, as well as for driving the inflammatory response in the tumor microenvironment. The increasingly common use of HA-conjugated drugs in oncology, as well as HA-based compounds as adjuvants in cancer treatment, adds further complexity to the understanding of the net effect of hyaluronan-CD44 activation in cancers. In this review, I focus on the significance of CD44 in malignancy and discuss the dichotomous function of the hyaluronan/CD44 axis in cancer progression.

## 1. Introduction

The stochastic model of carcinogenesis, which has dominated cancer research in the 20th century, suggests that all cells in a tumor population have an equal chance of acquiring and accumulating genetic or epigenetic mutations in crucial genes that regulate cell growth and differentiation [1]. This process leads to the emergence and selection of clones with advantages in proliferation, eventually enabling them to invade neighbouring tissues [2]. However, the idea that not all cells in a tumor are the same and only a specific subset possesses the capacity for self-renewal and the formation of new tumours—known as cancer stem cells (CSCs)—is gaining increasing support [3,4]. One of the well-established markers of CSCs is CD44, a multifunctional molecule that serves as a receptor for hyaluronic acid (HA) [5].

CD44 is a transmembrane glycoprotein expressed on the surface of a variety of cells, including cancer cells [6]. It has multiple isoforms (CD44v) resulting from alternative splicing, which contribute to its functional diversity [7]. HA, on the other hand, is an evolutionary conserved large polysaccharide component of the extracellular matrix (ECM) that is also expressed on the cell surface and inside cells and is abundant in various tissues [8]. In addition to serving as a structural framework, HA also activates intracellular signal transduction through its interaction with cell surface receptors, primarily CD44 [9].

The binding of HA to CD44 represents a critical molecular interaction that controls various physiological and pathological processes. HA binds to CD44 through a receptor-ligand interaction, as CD44 ectodomain includes an amino-terminal domain that contains an HA-binding “link module” motif. There is ample evidence that the hyaluronan/CD44 axis regulates several cellular functions including adhesion [10], migration [11], proliferation [12], and differentiation [13]. The interaction between HA and CD44 provides a structural scaffold for cell anchorage, allowing cells to adhere and migrate through the extracellular matrix during tissue development, wound healing, and immune responses [14,15]. Additionally, the hyaluronan/CD44 axis contributes to the regulation of inflammation [16], angiogenesis [17], metabolism [18], and stem cell maintenance [19,20]. Dysregulation of this axis has been implicated in various diseases [21], including cancer, where it promotes tumor progression, metastasis, and therapy resistance [22,23,24].

Given that HA is a naturally occurring polysaccharide found abundantly in the extracellular matrix and has high affinity for CD44 receptors, it is not surprising that HA-conjugated drugs have gained significant attention in cancer treatment, particularly due to their potential for targeted therapy and improved drug delivery [25,26,27]. However, this raises the question as to whether CD44 engagement by HA triggers downstream pathways that culminate in the acquisition of a more aggressive phenotype. For example, HA-based nanocarriers have been developed to improve drug delivery efficiency and reduce off-target accumulation [28]; however, these HA-based compounds could trigger CD44 signalling pathways in cancer cells, leading to unintended consequences such as the promotion of tumor growth, invasion, or metastasis. In principle, these outcomes may occur if the nanoparticles interact with CD44 receptors in a manner that stimulates pro-tumorigenic signalling cascades or promotes cancer cell survival and proliferation. This potentially harmful effect would also limit the use of HA concurrently with chemoradiation, a strategy that is increasingly used to prevent or reduce the onset of cancer-treatment-induced mucosal toxicity [29,30].

In this review, I will discuss the role of CD44 in cancer and highlight the potentially dichotomous function of the hyaluronan/CD44 axis in cancer treatment.

## 2. Hyaluronan Receptor CD44 as a Cancer Stem Cell Marker

The identification and characterization of CSCs have revolutionized our understanding of tumor biology and have significant implications for cancer treatment, particularly for overcoming therapy resistance [31]. CSCs are a small subpopulation of cells within tumours that possess stem-cell-like properties, including self-renewal capacity, multilineage differentiation potential, and resistance to conventional cancer therapies [4]. Interestingly, not all CSCs are equal, and distinct subpopulations exist that can lead to functionally different processes. Specifically, the expression of the hyaluronan receptor CD44 has been linked to the self-renewal capacity of CSCs and is associated with the maintenance and regulation of CSC populations [32]. Over the last decade, CD44 has emerged as a prominent marker for CSCs in various types of cancer and, hence, it provides valuable insights into tumor initiation, progression, and therapy resistance [23,33].

CD44, and particularly the CD44v isoform, functions as a marker for cancer stem cells (CSCs) not only due to its unique expression on the cell surface, but also, and more significantly, because of its potent role in regulating various CSC properties, partly via EGFR-mediated pathways [34]. These regulatory effects encompass a wide range of functions, including the integration and transmission of signals from the tumor environment and cellular surroundings to the cell’s nucleus, facilitating self-renewal [35]. Additionally, CD44 provides protective effects to CSCs by guarding against stress-induced damage or apoptosis caused by reactive oxygen species (ROS) or other detrimental stimuli [36,37].

CD44-positive CSCs exhibit enhanced tumorigenic potential, forming tumours more efficiently in xenograft models of different cancer types, compared to CD44-negative cells [38,39,40,41]. This suggests that CD44 is involved in the expansion of CSC populations in cancers, allowing for tumor growth and recurrence. Specifically, CD44 has been associated with the generation and maintenance of a subpopulation of cells with stem-like characteristics [42]. These CD44-positive CSCs possess a higher capacity for self-renewal, tumor initiation, and metastatic potential than their CD44-negative counterparts [43].

While these data suggest that CD44 is a key determinant of the phenotypic heterogeneity observed within tumours, it does not appear to reliably identify CSC populations in established cell lines [44], thus raising questions about the suitability of cell lines for the screening of CSC-specific therapies. This finding has major clinical implications, as phenotypic heterogeneity contributes to tumor complexity and poses challenges for effective treatment.

Mechanistically, CD44 promotes tumor growth via the activation of mitogen-activated protein kinases (MAPK) and phosphoinositide 3-kinase/protein kinase B (PI3K/AKT) signalling pathways and accelerates epithelial-to-mesenchymal transition (EMT) by activating AKT signalling, which results in the formation of EMT-associated recurrent tumours and apoptosis resistance [45]. Specifically, the HA/CD44 axis activates several oncogenic-signalling-pathways-associated cell surface receptors or domains, such as epidermal growth factor receptor (EGFR), c-Met, human epidermal growth factor receptor (HER) 2, transforming growth factor-beta receptor type 1 (TGFβR1), and non-receptor kinases (Src family) [6]. HA/CD44 interaction also activates ERM, ankyrin, Grb2, Gab-1, and Vav2, which drive cell migration via RAS, RhoA, and Rac GTPase families [6,46,47,48]. Whether these pathways are specific to CSCs or are, instead, activated in all CD44-positive cells it is not yet clear.

In summary, CD44 is often used as a marker for CSCs, particularly in breast cancer and in head and neck cancer, because CD44-positive cells are associated with properties that are often attributed to CSCs, such as self-renewal and tumor-initiating capabilities. However, it is important to point out the expression of CD44 is not universal among all CSCs and that CSCs are a heterogeneous population.

## 3. Clinical Significance of CD44 in Cancers

### 3.1. Expression of CD44 as a Prognostic Biomarker

CD44 expression in cancer has been correlated with both poor and favourable outcomes [49]; however, growing evidence now suggests that overexpression of CD44 and its isoforms is an unfavourable prognostic indicator in cancer patients [50,51,52,53,54,55,56,57,58,59,60,61]. A list of some of these studies, although not comprehensive, is provided in Table 1.

Recent meta-analyses have provided conclusive evidence for a predictive value of CD44 expression in cancer patients. For example, an analysis of nine studies, including 583 cases of pancreatic cancer, indicated that CD44 overexpression was predictive of poor five-year overall survival, more lymph node invasion, and a more-advanced stage, but was not associated with tumor size, differentiation, and distance metastasis [61].

Similarly, a meta-analysis assessing the prognostic significance of cancer stem cell markers in ovarian cancer from 52 studies concluded that CD44 correlated with worse disease-free survival, as well as with chemotherapy resistance [62].

Most recent studies also suggested that the CD44 isoforms such as CD44v3, CD44v6, and CD44v9, rather than receptor’s standard form (CD44s), harbour pro-tumorigenic potential. In one example, CD44v3+ cells, which represented a subpopulation of CD44+ cells, were detected in advanced preneoplastic lesions and presented CSCs chemoresistance and tumorigenic properties in vitro and in vivo [63]. The pooled analysis of 3918 colorectal cancer cases showed that CD44v6 overexpression was an independent prognostic marker of a lower five-year overall survival rate and was associated with more lymph node invasion and advanced stage [64].

CD44 overexpression seems to be a conserved mechanism in cancer progression, as a meta-analysis suggested that positive CD44v9 expression predicted a worse prognosis in most human cancers. While that article was retracted due to technical inaccuracies that affected the overall significance of the results, substantial primary evidence remains, indicating that CD44 variants, particularly CD44v9, are prognostic indicators of poor survival in patients with head and neck cancer [65], multiple myeloma [66], gastric cancer [67,68], upper-tract urothelial cancer [69], hepatocellular carcinoma [70], colorectal cancer [71], bladder cancer [72], pancreatic cancer [73], gallbladder cancer [74], and breast cancer [75]. Notably, CD44 can undergo isoform switching in tumor cells to support specific cellular functions. For example, the induction of EMT requires a switch in CD44v to CD44s isoform expression in breast cancer cells [45]. Other studies indicated that CD44v isoforms, rather than CD44s, were expressed in metastasis in several types of solid tumours and were associated with poorer prognosis [76].

In summary, previous studies showed that high levels of CD44 expression are associated with a number of factors that indicate a poorer prognosis, such as larger tumor size, more aggressive tumor grade, increased lymph node involvement, metastasis, and decreased survival. The role of specific variants and molecular switching is still poorly understood.

### 3.2. CD44 Polymorphisms and Cancer Risk

The function of CD44 in cancer is not only dependent upon CD44 expression levels, as recent studies demonstrated that many polymorphisms in CD44 were correlated with the risk of several types of cancer.

For example, in a cohort of 279 patients with lung adenocarcinoma, both CD44 rs713330 T/C and rs10836347 C/T polymorphisms exhibited significant associations with tumor size and invasion, independent of EGFR mutational status [77].

A quantitative meta-analysis, pooling the results of multiple studies to achieve increasing statistical power, provided multiple lines of evidence for the relationship between CD44 polymorphisms and cancer risk [78].

Another evidence-synthesis study based on 5788 cancer patients indicated that CD44 polymorphism rs13347 acts as a risk factor for cancer, especially in Chinese people, while the minor allele of polymorphism rs11821102 may be associated with a decreased susceptibility to cancer [79]. Together, these data showed that CD44 function, whether via upregulation or the expression of different phenotypes, is an important determinant in carcinogenesis.

### 3.3. CD44 and Resistance to Cancer Treatment

The onset of chemoresistance is one of the key challenges in cancer treatment and limits the clinical application of chemotherapy, as it contributes to tumor relapse, metastasis, and treatment failure.

Upregulation of CD44 has been associated with increased resistance to chemotherapy agents [80]. As cancer stem cells are known for their intrinsic resistance to chemotherapy and radiation, it is reasonable to expect that CD44-positive cells (CSCs) exhibit higher resistance to conventional therapies than CD44-negative cells (non-CSCs) within the tumor. Consistent with this view, CD44+ stem-like cells have been shown to be markedly resistant to paclitaxel and platinum treatment, two standard front-line therapeutics against epithelial tumours [81]. Accordingly, it has been proposed that only cells expressing CD44 on the surface persist after chemotherapy treatment and are able to rebuild the tumor afterwards [82]. The CSC niches may also be protected by the effects of chemotherapeutic drugs through HA.

Hyaluronan is a primary ECM component of the stem-cell niche and, similarly, HA-rich ECM provides a favourable microenvironment for the self-renewal and maintenance of CSCs [83]. Crucially, knockdown of CD44 decreases the adhesiveness of human colon cancer cells to HA, cancer-colony-forming ability, and xenograft tumorigenicity, while increasing susceptibility to etoposide-induced apoptosis [84]. Thus, the association between increased expression of CD44 and tumor aggressiveness (including chemoresistance) could simply reflect the role that the hyaluronan/CD44 axis plays in the maintenance and self-renewal capacity of CSC populations.

CD44 on the surface of cancer stem cells can also contribute to resistance via specific receptor-mediated and anti-apoptotic mechanisms. Chemoresistance can be mediated through the expression of P-glycoprotein (MDR1) coded by the multidrug resistance (MDR) gene, a cell membrane pump that either reduces drug uptake or causes efflux of the drug out of cancer cells. Recent data showed that CD44 increases the resistance of osteosarcoma cells to doxorubicin by upregulating P-glycoprotein expression [85]. The same study also identified differential regulation of several apoptosis-related genes in CD44-positive and -negative primary osteosarcomas [85]. This was in agreement with the current view that CD44 plays an indispensable role in activating survival pathways that protect cancer cells from apoptosis [32], as described in the following paragraph.

## 4. Mechanistic Basis for the Role of Hyaluronan/CD44 Axis in Cancer

**Figure 1:**
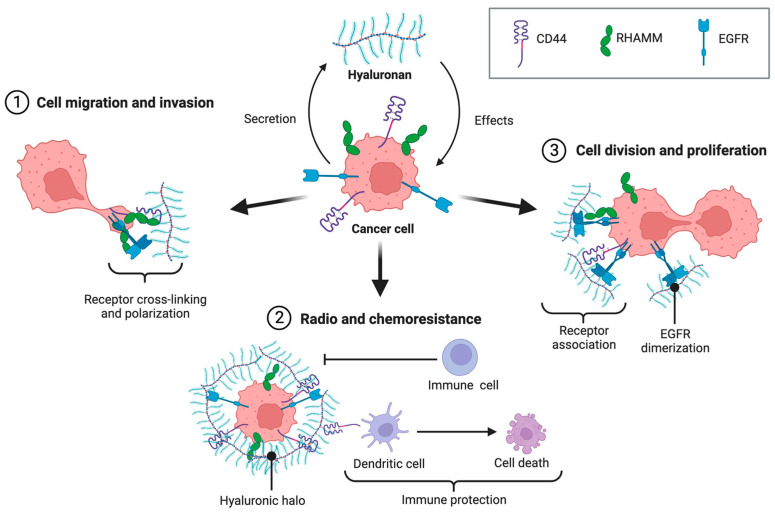
Hyaluronan impacts cancer cells by interacting with its receptors, CD44 and RHAMM, which in turn interact with other receptors, such as EGFR. This causes increasing migration, invasion, and proliferation, as well as radio- and chemotherapy resistance. Moreover, cancer cells secrete HA, which forms a halo that induces dendritic cell death and acts as an immunoprotective barrier.

The interaction between HA and its receptors is involved in several key processes that contribute to cancer development and progression, such as cell adhesion, migration, proliferation, differentiation, survival, and chemoresistance (Figure 1).

The binding of HA to CD44 can lead to the formation of HA/CD44 complexes on the cell membrane, and these complexes can initiate intracellular signalling pathways. Extracellularly, the binding of HA to CD44 allows cells to adhere to and interact with the extracellular matrix, as well as with neighbouring cells. Together, these processes controlled by the hyaluronan/CD44 axis are important for carcinogenesis. First, CD44 interacts with HA in the extracellular matrix, promoting cancer cell adhesion and invasion, which can shield cancer cells from the cytotoxic effects of chemotherapy drugs. Second, it plays a role in the evasion of apoptosis, as CD44 overexpression can activate anti-apoptotic pathways, allowing cancer cells to survive chemotherapy-induced cell death. Additionally, CD44 can influence drug-efflux mechanisms, such as upregulating drug transporters, thereby reducing intracellular drug concentrations.

These key events are mediated through the activation of intracellular signalling pathways, including protein kinases, GTPases, and transcription factors. In one example, CD44 engagement can lead to the activation of Src, a non-receptor protein tyrosine kinase, which is often associated with the regulation of cell adhesion and motility. Src phosphorylates various target proteins, including other kinases and adaptor proteins, in response to CD44 activation. One of the downstream targets of Src is the activation of the ERK pathway. Src can activate the Ras protein, which in turn activates the Raf-MEK-ERK cascade. This signalling cascade ultimately leads to the activation of ERK. Activated ERK translocates to the nucleus, where it can phosphorylate transcription factors, leading to changes in gene expression. These changes in gene expression can influence various cellular processes; in the context of CD44 signalling, this can include the regulation of cell adhesion, migration, and proliferation, which are crucial in processes like tissue remodelling, wound healing, and cancer metastasis.

Binding of hyaluronan to CD44 results in the direct or indirect interaction of CD44 with signalling receptors, such as ErbB2, EGFR, and TGF-beta receptor type I, and can also lead to interaction with non-receptor kinases of the Src family or Ras family GTPases [47]. The mechanisms of regulation of these interactions in different tumor cell types and cancer stages are not well understood and may involve specific variants of CD44. In the following paragraphs, I will explore the CD44-dependent molecular mechanisms regulating key cellular functions that are thought to be important in cancer pathophysiology.

### 4.1. Cell Adhesion and Migration

One mechanism by which the hyaluronan/CD44 axis promotes cancer is through its involvement in cell adhesion and migration. CD44 is capable of binding to HA, which provides a scaffold for cancer cells to adhere to and migrate through the extracellular matrix. This interaction promotes tumor invasion and metastasis, facilitating the spread of cancer cells to distant sites. Additionally, HA/CD44 binding can induce signalling cascades that promote cytoskeletal rearrangements and enhance cell motility.

Previous research showed that the glycosylation of CD44, rather than its overall expression, controls the adhesion of cancer cells to HA, as well as the extent and affinity of this binding [86]. This may help explain some contrasting results relative to the expression of CD44 in tumours. Intracellularly, CD44 interacts with a number of membrane-associated cytoskeletal proteins and Rho GTPases, and this interaction causes cytoskeleton activation and results in several important HA-mediated functions such as cell adhesion, proliferation, and migration [87].

The pro-migratory mechanisms of HA/CD44 are likely different in epithelial and mesenchymal cells and, hence, in carcinomas vs. sarcomas. In one example, the apoptosis-stimulating protein of p53 (iASPP) was shown to physically interact with CD44s (but not with the variant isoform CD44v) following hyaluronan stimulation in fibroblasts, but not in epithelial cells, thereby promoting hyaluronan-induced CD44-dependent migration and adhesion of fibroblasts [88]. Data also suggested that high-molecular-weight hyaluronic acid (HMW-HA) reduces cell migration, while low-molecular-weight hyaluronic acid (LMW-HA) increases it in cells of mesenchymal origin [89].

In epithelial cells, mice with epidermal-specific inactivation of Cd44 display reduction in epidermal stiffness and delayed wound healing, thus confirming the role of CD44 in cell adhesion and migration [90]. CD44/hyaluronan interaction can activate intracellular signalling pathways, such as focal adhesion kinase (FAK) and Src, which regulate cytoskeletal rearrangements and facilitate cell motility [47].

A key mechanism involved in the acquisition of a migratory and metastatic phenotype is the so called epithelial-to-mesenchymal transition. CD44s accelerates EMT by activating AKT signalling, which results in the formation of EMT-associated recurrent tumours and apoptosis resistance [45]. The EMT signalling triggered by CD44 is likely to be variant-dependent: for example, CD44s, but not CD44v, regulates the TGF-β-signalling-mediated mesenchymal phenotype [91]. The reverse is also true, as TGF-β1 regulates CD44 splicing toward CD44s expression via the RNA binding protein PCBP1, thus promoting EMT and prostate-cancer-cell migration, invasion, and tumor initiation [92].

In summary, the hyaluronan/CD44 axis is important in the acquisition of a migratory and invasive phenotype. On the basis of the data presented here, it is perhaps not surprising that CD44 is regarded as a well-established pro-metastatic gene in several malignancies [6].

### 4.2. Cell Survival and Proliferation

The hyaluronan/CD44 axis also influences cancer cell survival and proliferation. CD44 activation by HA can trigger pro-survival signals, promoting cell survival and protecting cells from apoptosis, and it also contributes to cell proliferation.

Knockdown of CD44 was found to inhibit the proliferation, migration, and invasion of CVCs and to induce apoptosis, promote cell-cycle arrest at the G1/G0 phase in vitro, and suppress in vivo tumorigenesis and metastasis of CSCs [93].

Mechanistically, CD44 activation has been associated with the activation of various oncogenic signalling pathways, including the PI3K/AKT and MAPK/ERK pathways, which regulate cell proliferation, survival, and tumor growth [94]. In one example, it was shown that CD44 functions as an upstream regulator, sensing the extracellular environment to modulate the ERK, AKT, and Hippo-YAP pathways that cooperatively control downstream gene expression to modulate cell-contact inhibition of proliferation, cell cycle progression, and maintenance of tumor-initiating cells [95].

In several types of cancer cells, the binding of hyaluronan to CD44 results in direct or indirect interaction of CD44 with receptors that regulate oncogenic pathways, such as ErbB2, EGFR, and TGF-beta receptor type I, and can also lead to interaction with non-receptor kinases of the Src family or Ras family GTPases [96]. CD44 is also a major Wnt target gene in the intestine, and it is essential for Wnt-induced tumor progression in colorectal cancer [97].

Taken together, these data show that CD44 is instrumental in the acquisition and maintenance of cancer hallmarks by sustaining proliferative signalling and evading apoptosis.

### 4.3. Therapy Resistance Mechanisms 

The anti-apoptotic and pro-survival signals discussed above can also promote cancer cell resistance to cell death induced by chemotherapy agents. Other therapy resistance mechanisms triggered by the hyaluronan/CD44 interaction include its ability to regulate drug-efflux pumps and to modify the microenvironment. Overall, the activation of CD44 signalling enables resistance of CSCs to cytotoxic treatments.

The intracellular concentration of many anti-cancer medications such as tyrosine kinase inhibitors, as well as natural products such as daunorubicin, actinomycin D, vinblastine, doxorubicin, vincristine, etoposide, and paclitaxel, is regulated by membrane transporters of the multidrug resistance (MDR) family [98].

A previous study showed that CD44 increases the resistance of osteosarcoma cells to doxorubicin by upregulating the levels of MDR1 protein expression, and that deletion of CD44 leads to doxorubicin-dependent p53 activation and a profound Perp upregulation [85].

CD44/HA signalling mediates resistance to a wide range of chemotherapeutic drugs [98], including PI3Kα inhibitors. Specifically, the interaction of CD44 with HA initiates the Src-ERK signalling cascade, which subsequently maintains AKT and mTOR activity in the presence of a PI3Kα inhibitor [99]. Crucially, disruption of the CD44/HA interaction prevented the activation of this pathway, which in turn restored sensitivity to the chemotherapeutic agent [100]. In head and neck cancer, CD44 was shown to contribute to chemoresistance and to upregulate expression of the MDR pump via miR-21, by activating the stem cell marker Nanog [101]. Additionally, drug resistance can be activated downstream of CD44 through the Stat3 pathway in several other carcinomas, including breast [80] and ovarian [102] cancer cells.

Importantly, the CD44 activation of specific survival and drug-resistance pathways is highly dependent on microenvironmental cues [49], prompting an appraisal of the role of the tumor microenvironment in the context of CD44 signalling.

### 4.4. Tumour Microenvironment

The hyaluronan/CD44 axis can modulate the tumor microenvironment, and this influences cancer progression and chemoresistance. HA accumulation in the tumor stroma, mediated by CD44, creates a supportive niche for cancer progression [103]. Increased HA deposition promotes tumor angiogenesis, immune evasion, and inflammation, facilitating tumor growth and metastasis [104]. Moreover, HA can act as a reservoir for growth factors and cytokines, influencing various cellular processes in the tumor microenvironment.

HA/CD44 interaction has implications in inflammation (both pro- and anti-inflammatory roles), as well as for immune responses. For example, CD44 is involved in leukocyte recruitment and activation during inflammatory processes [105]. The binding of HA to CD44 on immune cells can modulate their functions, including cytokine production, cell migration, and immune-cell activation. This interaction can also suppress the activation of T cells, which can lead to tolerance against neoplastic antigens.

It is important to note that different microenvironmental factors affect CD44 activation. For instance, while low-molecular-weight hyaluronic acid (LMW-HA) in the tumor microenvironment promotes tumorigenesis, abundant high-molecular-weight hyaluronic acid (HMW-HA) polymers also provide a stable source for generating LMW-HA in the microenvironment [106], making it difficult to establish their precise contribution to tumorigenesis. In another example, hyaluronan oligomers were shown to inhibit anchorage-independent growth of tumor cells in vitro and cancer growth in vivo by suppressing the phosphoinositide 3-kinase/Akt cell survival pathway in mammary carcinoma cells [107]. Thus, CD44 activation is highly dependent on microenvironmental cues.

Overall, the literature presented here supports the role of the hyaluronan/CD44 axis in the acquisition of a migratory, metastatic, and chemotherapy-resistant phenotype, while also highlighting the crucial role of the microenvironment in enabling the cancer-promoting function of CD44.

## 5. The Use of Hyaluronic Acid in Oncology: Is It Safe?

The identification and targeting of CD44-positive CSCs hold promise for improving cancer treatment outcomes. Strategies aimed at disrupting CD44 signalling pathways or targeting CD44-positive CSCs specifically have been investigated. These include the use of monoclonal antibodies, small molecule inhibitors, RNA interference-based approaches, and immunotherapies [25].

Targeting CD44-positive CSCs not only aims to eliminate the cells responsible for tumor growth and recurrence, but also disrupts the microenvironment and signalling networks that support CSC maintenance and therapy resistance. In this regard, an interesting use of HA is as a drug conjugate [108]. As reviewed in the previous paragraphs of this article, CD44 receptors are often overexpressed on the surface of cancer cells and, specifically, on tumor-promoting CSCs. By conjugating anticancer drugs with HA, researchers were able to exploit this specific interaction for targeted drug delivery to cancer cells.

A puzzling aspect of many studies is the characterization of hyaluronan-induced oncogenic signalling. Although these studies resulted in apparently solid data that were indicative of the pro-tumorigenic effects of CD44 activation, they are difficult to reconcile with the long history of safe utilization of hyaluronan in numerous reconstructive or regenerative capacities in human patients [47]. More recently, hyaluronan-based hydrogels were developed for a variety of purposes, including drug delivery, encapsulation of progenitor cells, and tissue engineering [47,109]. The wide use of exogenous HA in medicine suggests that the oncogenic effects of hyaluronan only occur in the context of the tumor microenvironment and that the stromal hyaluronan and/or the tumor-cell-produced hyaluronan play an important role in tumorigenesis [110]. This still poses some potential risks for HA use in oncology, as discussed below.

### 5.1. Hyaluronan-Conjugated Drugs 

Hyaluronic acid (HA)-conjugated drugs are used in oncology for various purposes, primarily to improve drug delivery and target cancer cells more effectively. The use of these drugs offers several advantages in cancer treatment [111,112], as summarized in Table 2.

First, HA acts as a targeting moiety, allowing the drug to selectively bind to and enter cancer cells that have an elevated CD44 expression. This focused strategy decreases unintended effects on other tissues and enhances therapeutic effectiveness by concentrating the drug precisely at the tumor site, while limiting its exposure to healthy tissue. Additionally, drugs conjugated with HA can overcome biological obstacles that are present in tumours, such as the dense extracellular matrix, by leveraging HA’s affinity for its receptor. This enables deeper penetration into the tumor tissue, ultimately boosting drug accumulation at the intended target site [113].

HA possesses inherent biocompatibility, biodegradability, and low immunogenicity, making it an attractive carrier for drug-delivery systems. The hydrophilic nature of HA promotes prolonged circulation time in the bloodstream, reducing rapid clearance and improving drug availability [114]. Additionally, HA can encapsulate or bind various types of anticancer agents, including chemotherapy drugs, small molecules, proteins, and nucleic acids, enabling a versatile platform for drug delivery [108].

HA-linked drugs have been utilized within a range of delivery platforms, including nanoparticles, micelles, liposomes, and hydrogels. These utilizations aim to enhance factors such as the timing of drug release, stability, and the efficiency of targeting tumours. Moreover, these delivery systems offer the flexibility for further enhancements, such as the integration of responsive release mechanisms, imaging agents, or ligands, to improve cellular uptake or targeting precision [115].

A still unsolved question relates to the possible tumor-promoting effects of CD44/hyaluronan engagement and, hence, to the safety of HA-conjugated drugs. One of the key concerns is based on the observation that shorter bioactive HA fragments (e.g., LMW-HA) can interact with cancer cells and alter their activity in a different manner than that of HMA-HA [116]. Specifically, while HMW-HA maintains tissue homeostasis, HA breakdown products generated by hyaluronidases or ROS are often associated with enhanced invasion of cancer cells and tumor growth [117]. While current evidence does not suggest that this is the case for HA-conjugated drugs, ad hoc studies should be designed to rule out this possibility.

### 5.2. Effect of Exogenous Hyaluronan

In addition to providing a versatile platform for anticancer drug delivery, HA is often administered in cancer patients and/or concurrently with chemotherapeutic agents for a number of reasons, including the prevention of cancer-treatment-associated mucosal toxicities [29,118].

Some evidence demonstrates that high HA levels lead to poorer chemotherapy treatment outcomes. Early studies in breast cancer patients showed that lower HA concentrations were observed in patients responding to chemotherapy; however, the initial level of serum HA had no predictive value for responses to chemotherapy [119].

More recently, baseline plasma HA was shown to be reliably associated with bone metastasis and to predict poor prognosis in lung cancer patients [120]; however, it is not known whether high levels of plasma hyaluronan are a consequence, rather than a cause, of metastasis. Another interesting report raised the possibility that cancer cells synthesize HA in an attempt to protect the cancer from chemotherapy [121]. Cancer-derived HA could, therefore, participate in the acquisition of chemoresistance. This is not entirely unexpected, given that HA is effectively used in cancer patients to reduce the chemoradiation-induced toxic effects triggered by oxidative stress in healthy tissues [122,123], but in doing so it might also interfere with the pro-oxidant mechanisms that underlie the efficacy of antineoplastic treatments that target cancer cells.

Mechanistically, it has been shown that HA mediates the formation of a complex including CD44 and the epidermal growth factor receptor (EGFR), which plays major roles in chemoresistance in head and neck squamous cell carcinoma (HNSCC) [124]. In ovarian cancer, carboplatin chemotherapy induces HA production, which contributes to chemoresistance by regulating ABC transporter expression [121]. This suggests that the HA/CD44 signalling pathway, rather than CD44 as a molecule, could be a promising target in resistant cancers.

Exogenous administration of HA can also potentially increase the bioavailability of LMW-HA via activity of hyaluronidases. Importantly, previous studies suggested that LMW-HA may have pro-tumorigenic effects. LMW-HA accumulation is associated with tumor aggressiveness in that it triggers the expression of specific cytokines and proteases that are required for remodelling of the tumor microenvironment [125,126]. More broadly, LMW-HA has been associated with promoting cell proliferation, migration, invasion, angiogenesis, and tissue remodelling, which can be factors in tumor growth and progression [127].

LMW-HA can also participate in pro-inflammatory responses—for example, by activating immune cells, such as macrophages, and by promoting the release of pro-inflammatory cytokines [128]. This contributes to local inflammation, which may play a role in various pathological conditions, including cancer. In one example, LMW-HA was found to attract macrophages that polarized into immunosuppressive subpopulations, which protect tumor cells from adaptive immune-cell killing [129]. It is important to note that LMW-HA has high affinity for the receptor for hyaluronan-mediated motility (RHAMM), which can contribute to cell-signalling pathways that are associated with cancer.

Hence, the relationship between HA and its effects on tumorigenesis and inflammation is complex and context-dependent. These inconsistencies arise partly due to variations in the utilization of cell lines and animal models, differences in treatments, varying culture conditions, and other experimental factors. However, these discrepancies fundamentally mirror the dual nature of this molecule and its role as a matrix-sensing entity.

It is clear that the use of HA would be unjustified if it caused the growth of tumor cells or inhibited the antineoplastic effects of cancer treatment. This concern was partly addressed in a mouse model of ovarian carcinogenesis, where conjugation to HA with SN-38 (the active metabolite of irinotecan) significantly improved the profile of in vivo tolerability and enhanced therapeutic efficacy for ovarian cancer treatment. Further studies also reported that HA derivatives and different types of HA-drug conjugates have potential synergistic antitumor effects.

In summary, the dual role of HA in cancer is complex and multifaceted. Pro-tumorigenic mechanisms of HA include enhanced tumor growth, cell proliferation, invasion, and metastasis, whereas potential tumor suppressor roles include immune system activation and inhibition of angiogenesis. It is important to note that the effects of HA in cancer are highly context-dependent. Factors such as HA molecular weight, concentration, tumor type, and the presence of specific HA receptors on cancer cells can influence whether HA has a promoting or inhibitory effect on cancer progression. The use of HA-conjugated drugs represents a promising strategy in cancer treatment, while at the same time demonstrating the safety of this molecule. By harnessing the targeting ability of HA and its interaction with CD44 receptors, these drug delivery systems offer improved specificity, enhanced tumor accumulation, and reduced side effects.

## 6. Conclusions

The mechanistic basis for the role of the hyaluronan/CD44 axis in cancer involves its contribution to cell adhesion, migration, survival, proliferation, CSC maintenance, and modulation of the tumor microenvironment. While targeting this axis represents a potential therapeutic strategy to overcome cancer progression and treatment resistance, further research is needed to fully understand the complex mechanisms underlying CD44-mediated pro-tumorigenic signalling and whether targeting the hyaluronan/CD44 axis ultimately improves patient outcomes in cancer therapy. Given the dual role of HA in cancer, continued research and development in this field is warranted to ensure that HA-based compounds do not hamper the effectiveness of cancer treatment.

## Figures and Tables

**Table 1:** Expression of CD44 in different cancer types.

Article [Ref]	Cancer Type	Main Findings
Wang et al., 2019 [50]	Colorectal	CD44, CD44v6, or CD44v2 correlated with unfavorable overall survival; CD44 predicts poor differentiation, lymph node metastasis, and distant metastasis.
Razmi et al., 2021 [51]	Gastric	The expression of CSC markers was mostly associated with worse outcomes in patients with GC, both overall and individual.
Mare et al., 2021 [52]	Rectal	Elevated levels of CD133, CD44, ALDH1, Lgr5, and G9a were associated with RT resistance and poor prognosis.
Abdoli Shadbad et al., 2021 [53]	Breast	CD44 and CD44^+^CD24^−/low^ phenotypes were associated with inferior prognosis in breast cancer patients.
Fahmi et al., 2021 [54]	Cervical	Increased expression of CD44 was associated with poor overall survival in cervical cancer tissues.
Zhang et al., 2015 [55]	Osteosarcoma	CD44V6 over-expression was associated with overall survival rate and metastasis in osteosarcomas.
Luo et al., 2014 [56]	Non-small cell lung cancer	Overexpression of CD44-v6 was significantly associated with tumor differentiation, tumor histological type, clinical TMN stage, and lymph node metastasis.
Chen et al., 2014 [57]	Head and Neck	CD44 was related to worse T category, N category, tumor grade, and prognosis in pharyngeal and laryngeal cancer, but not in oral cancer.
Al-Mosawi et al., 2020 [58]	Oesophageal	CD44 overexpression negatively correlated with five-year overall survival and was associated with lymph node metastasis, vascular invasion, and recurrence.
Chai et al., 2014 [59]	Pharyngolaryngeal cancer	High expression of CD44-v6 was related to a poor five-year OS rate and was associated with tumor size, lymph node metastasis, and poor prognosis.
Li et al., 2015 [60]	Renal cell carcinoma	High CD44 expression correlated with high Fuhrman grade, recurrence, MVI, and poor prognosis.
Liu et al., 2018 [61]	Pancreatic cancer	CD44 overexpression was associated with poor five-year overall survival rate, more lymph node invasion, a more-advanced T stage, and a more-advanced TNM stage.

**Table 2:** Summary of the advantages of hyaluronic acid (HA)-conjugated drugs in oncology.

Use	Brief Description of HA-Conjugated Drugs
Targeted Drug Delivery	Designed to specifically target cancer cells that overexpress CD44 receptors, which bind to HA. This targeted approach can enhance drug delivery to cancer cells while reducing off-target effects.
Reduced Systemic Toxicity	Targeted delivery of HA-conjugated drugs can reduce systemic toxicity because the drugs primarily accumulate in tumor tissues, sparing healthy cells.
Improved Pharmacokinetics	Conjugating drugs with HA can alter their pharmacokinetics, making them more suitable for cancer therapy. This can include increasing drug stability and circulation time in the bloodstream.
Enhanced Drug Efficacy	Improve the efficacy of chemotherapy or other therapeutic agents by ensuring their delivery to the tumor site and enhancing their internalization by cancer cells.
Combination Therapy	Can be used in combination with other cancer therapies, such as immunotherapy or radiation therapy, to enhance their effectiveness.
Treatment of Drug-Resistant Cancers	In some cases, HA-conjugated drugs may help overcome drug-resistance mechanisms in cancer cells, as the targeted approach can make it more difficult for cells to evade treatment.
Diagnostic and Imaging Agents	Can be used for diagnostic purposes, helping to visualize tumours or assess disease progression.
Potential for Personalized Medicine	HA conjugates have the potential for personalized medicine approaches, as they can be designed to target specific molecular characteristics of a patient’s cancer.

## Data Availability

No new data were generated for this article.
